# IL-17A, IL-22, IL-6, and IL-21 Serum Levels in Plaque-Type Psoriasis in Brazilian Patients

**DOI:** 10.1155/2015/819149

**Published:** 2015-08-13

**Authors:** Priscilla Stela Santana de Oliveira, Pablo Ramon Gualberto Cardoso, Emerson Vasconcelos de Andrade Lima, Michelly Cristiny Pereira, Angela Luzia Branco Pinto Duarte, Ivan da Rocha Pitta, Moacyr Jesus Barreto de Melo Rêgo, Maira Galdino da Rocha Pitta

**Affiliations:** ^1^Laboratório de Imunomodulação e Novas Abordagens Terapêuticas (LINAT), Centro de Ciências Biológicas, Núcleo de Pesquisa em Inovação Terapêutica Suely Galdino (NUPIT-SG), Universidade Federal de Pernambuco (UFPE), Avenida Prof. Moraes Rego, 1235, Cidade Universitária, 50670-901 Recife, PE, Brazil; ^2^Hospital das Clínicas, Universidade Federal de Pernambuco (UFPE), Avenida Prof. Moraes Rego, 1235, Cidade Universitária, 50670-901 Recife, PE, Brazil

## Abstract

Psoriasis is a chronic inflammatory skin disease characterized by alterations in cytokines produced by both Th1 and Th17 pathways. The aim of this study was to evaluate serum levels of pivotal cytokines and correlate them with clinical parameters. Serum samples from 53 psoriasis patients and 35 healthy volunteers, matched by the proportion of sex and age ratios, were collected for ELISA cytokine detection. Psoriasis Area and Severity Index (PASI) was assessed at the time of sampling in psoriasis patients. Our findings demonstrate that IL-17A, IL-22, and IL-6 serum concentrations were significantly higher in psoriasis patients than in the control group. No statistical correlation could be found between cytokines concentrations, PASI score, and age in this study. Although our results do not show any correlation between serum levels of IL-17A, IL-22, and IL-6 and disease activity, the present study confirms that they were increased in Brazilian psoriasis patients in comparison to healthy volunteers.

## 1. Introduction

Psoriasis is a chronic inflammatory skin disease that can be associated with other systemic disorders like cardiovascular disease, metabolic syndrome, and inflammatory bowel disease [1, 2]. The financial and psychological impacts lead to anxiety and depression especially in individuals with active professional and social lives [3–5]. The predominant clinical presentation of psoriatic lesions is characterized by the formation of scaly, well-demarcated erythematous plaques due to hyperproliferation of keratinocytes [6, 7].

*In vitro* models studies have revealed a complex interaction of dendritic cells, epidermal keratinocytes, and infiltrated immune cells and their proinflammatory cytokines [8, 9]. In the past decade, Th1 cytokines such as interferon-*γ* (IFN-*γ*) and tumor necrosis factor-*α* (TNF-*α*) were considered to play a major role in this disease [10], but recent evidence points toward a central role of IL-23 and IL-17A in the physiopathogenesis of psoriasis [11–13].

IL-17A enhances the expression of S100 proteins, chemokines CCL20, CXCL1, CXCL3, CXCL5, CXCL6, and CXCL8, and VEGF in keratinocytes leading to aberrant cell differentiation, proliferation, and immune activation [14–16]. IL-22 decreases the expression of CK10, filaggrin, and involucrin and induces the production of matrix metalloproteinases 1 and 3 (MMP1 and MMP3), which facilitate the infiltration of immune cells and the restructuring of the epidermis [17, 18]. In mouse models of psoriasis elevated levels of IL-21 are associated with CK6 and CK16 overexpression, consistent with an abnormal keratinocyte proliferation [19]. Finally, IL-6 can induce IL-1*α* expression, which promotes keratinocyte proliferation and the activation of NF*κ*B and C/EBP*β* transcription factors [20]. Besides,* in vitro* combination of IL-6 and TGF-*β* drives the differentiation of CD4^+^ T naïve cells into a Th17 phenotype [21].

This study was designed to evaluate the expression of IL-17A, IL-22, IL-21, and IL-6 in serum samples from northeastern Brazilian patients with plaque-type psoriasis and its correlation with disease activity.

## 2. Materials and Methods

### 2.1. Population under Study

Serum samples from fifty-three patients with plaque-type psoriasis attending the Dermatology and Rheumatology Outpatient Clinic at Universidade Federal de Pernambuco, Recife, Brazil, and thirty-five healthy donors were collected. The Human Ethics Committee of the UFPE approved the study protocol (CEP-CCS 528/11). Blood samples obtained by venipuncture were centrifuged at 2000 rpm for 10 minutes. The serum was collected and stored at −80°C. Formal written consent was obtained from all the patients and healthy volunteers enrolled in the study.

### 2.2. Clinical Assessment

Only patients with a diagnosis of plaque-type psoriasis in strict accordance with the diagnostic criteria of Nestle et al. [6] were included in the study. Psoriasis Area and Severity Index (PASI) [22] assessment was used to grade the disease activity of patients with psoriasis at the time of blood collection as mild (0–10), moderate (11–20), and severe (>20) [23]. Patients with other coexistent autoimmune disorders, acute or chronic infections, and malignancies, receiving systemic treatment, immunosuppressive drugs, or phototherapy, were excluded from our study. Patients were off topical treatment for 4 weeks prior to the PASI score evaluation and blood sample collection.

### 2.3. Measurements of Cytokines

Serum levels of IL-17A, IL-6, IL-21, and IL-22 were measured by using enzyme-linked immunosorbent assay (ELISA) kits (eBiosciences, USA, and BD Biosciences, USA) according to the manufacturers' instructions. The absorbance used was the difference between 570 and 450 nm readings. The minimum limits of detection of the ELISA kits used in the experiment were 3.9 pg/mL for IL-17A, 4.69 pg/mL for IL-6, 8 pg/mL for IL-21, and 15.63 pg/mL for IL-22.

### 2.4. Statistical Analysis

Statistical analyses were performed by GraphPad PRISM 6.01 software. The D'Agostino-Pearson and Mann-Whitney tests were used. Results were expressed as median and interquartile range or mean ± standard deviation (SD) for variables with normal distribution. Pearson's correlation coefficient was used in correlation analyses. The statistical significance was accepted when *p* < 0.05.

## 3. Results

### 3.1. Clinical and Laboratory Values of Psoriatic Patients

Clinical and demographic characteristics of patients and controls are detailed in Table 1.

### 3.2. IL-17A, IL-22, IL-6, and IL-21 Serum Levels in Psoriatic Patients and Healthy Controls

The cytokines investigated in this study were detected in serum samples from all patients whereas IL-17 and IL-6 were below the minimum detection level of the kit in healthy controls. According to the D'Agostino-Pearson test, all results obtained with the dosage of IL-17, IL-22, IL-6, and IL-21 do not follow the normal distribution and these cytokines were expressed by median with interquartile range (Table 2).

IL-17A, IL-22, and IL-6 serum concentrations were significantly higher in psoriatic patients than in healthy donors. In contrast, IL-21 levels were numerically higher in healthy controls than in patients, but the difference was not statistically significant.

### 3.3. Correlations between IL-17A, IL-22, IL-6, and IL-21 Serum Levels of Psoriatic Patients and PASI Score and Age

In our study we observed that both IL-21 levels tended to correlate with Psoriasis Area and Severity Index, but no statistical significance was shown (*p* = 0.0952). Serum levels of IL-17A and IL-6 were not correlated with PASI. No correlation was found between serum levels of the cytokines analyzed in this study and severity grading or age of patients.

## 4. Discussion

Our study revealed increased IL-17A serum levels in psoriasis patients in comparison with healthy controls, but no statistical correlation between this cytokine and PASI or age was found. These results are in agreement with a randomized controlled trial that showed high blood levels of IL-17A in psoriasis patients before treatment with etanercept and acitretin [24]. More recently, a transcriptome evaluation study also demonstrated high IL-17A serum levels in moderate-severe psoriasis patients [25].

In another study, Takahashi et al. [26] determined serum levels of diverse cytokines and growth factors in Japanese patients with psoriasis, including some cases with psoriasis guttata, erythrodermic psoriasis, and psoriatic arthritis. They observed that IL-17 levels were higher than those in healthy controls and strongly correlated with PASI.

In contrast with our findings in serum samples, significantly increased mRNA expression and protein accumulation of these cytokines have been found in psoriatic skin biopsy specimens [27–29].

We did not find a statistically significant correlation between the IL-22 serum concentration and disease activity. In a recent study, Michalak-Stoma et al. [30] found significantly higher serum concentrations of IL-22 in psoriatic patients in comparison with healthy controls and a significant positive correlation with psoriasis severity measured by PASI and BSA score. The correlation they found may be due to the greater range of PASI values in their patients (4.8 to 64.2) compared to that in our study (7 to 41). Their study population also differs from ours in the higher proportion of men (80%).

Some studies have shown increased serum levels of IL-21 in psoriasis patients with respect to healthy controls [31, 32]. However only He et al. [32] found a statistically significant positive correlation with PASI.

In our experiments we found elevated serum IL-6 levels in psoriatic patients with no correlation with disease severity, as has been reported before [25, 26, 29]. In a recent study Cordiali-Fei et al. [33] showed increased IL-6 serum levels in patients before biological therapy, but no correlation with PASI was attempted. Elango and colleagues reported a positive correlation with only two components of PASI, namely, infiltration and desquamation [34].

As previously discussed, cytokines well described in psoriasis pathogenesis are also elevated in northeastern Brazilian patients' serum. IL-17 is able to induce pivotal chemokines involved in development and amplification of psoriasis plaque [14–16]. IL-6 is associated with pustular variants of disease in which it promotes keratinocyte release of neutrophil chemoattractant factors [35]. Finally, according to Sa et al. and Wolk et al., IL-22 inhibits the final stages of keratinocyte differentiation leading to absence of a granular layer and persistence of cell nuclei in the superficial cells [36, 37].

## 5. Conclusion

Our study revealed increased IL-17A serum levels in Brazilian psoriasis patients in comparison with healthy controls, but no statistical correlation between this cytokine and PASI or age was found.

## Figures and Tables

**Table 1:** Clinical features of psoriasis patients and healthy controls.

Characteristics	Mean (range or SD)	Mean (range or SD)
	Patients (*n* = 53)	Controls (*n* = 35)
Female	24	16
Male	30	19
Age (years)	50.2 ± 13.3	46.0 ± 11.0
PASI (*n* = 53)	16.4 (7–41)	—
Mild (*n* = 16)	8.3 ± 1.1	—
Moderate (*n* = 21)	15.2 ± 3.0	—
Severe (*n* = 16)	28.5 ± 4.8	—

**Table 2:** Serum cytokines in psoriasis patients and healthy controls.

Cytokine (pg/mL)	Patients				Control	Man-Whitney test
	Mild (*n* = 16)	Moderate (*n* = 21)	Severe (*n* = 16)	All groups (*n* = 53)	(*n* = 35)	
IL-17	3.9 (3.9–2420)	3.9 (3.9–3530)	19.55 (3.9–3170)	**3.9** (3.9–3530)	**3.9** (3.9-3.9)	<0.0001^*∗∗∗∗*^
IL-6	4.6 (4.6–203.3)	4.6 (4.6–469.5)	4.6 (4.6–11.5)	**4.6** (4.6–469.5)	**7.8** (7.8–57.2)	<0.0001^*∗∗∗∗*^
IL-22	7.81^+^	7.8 (7.8–46.3)	7.8 (7.8–103.9)	**7.8** (7.8–103.9)	**4.6** (4.6-4.6)	0.0247^*∗*^
IL-21	15.6 (15.6–195.5)	15.6 (15.6–230.5)	23 (7.8–335.5)	**15.6** (15.63–335.5)	**66.33** (7.8–1621.3)	0.2516

Values are represented by median with (minimum–maximum).

^+^Samples which have no variation.

^*∗*^ *p* < 0.05 and ^*∗∗∗∗*^ *p* < 0.0001.

## Abbreviations

C/EBP*β*: CCAAT-enhancer-binding proteins *β*
CCL: Chemokine (C-C motif) ligand
CK: Cytokeratin
CXCL: Chemokine (C-X-C motif) ligand
ELISA: Enzyme-linked immunosorbent assay
ESR: Erythrocyte sedimentation rate
IFN-*γ*: Interferon-*γ*
MMP: Matrix metalloproteinases
NF*κ*B: Factor nuclear kappa B
PASI: Psoriasis Area and Severity Index
TNF-*α*: Tumor necrosis factor-*α*
VEGF: Vascular endothelial growth factor.
